# Alterations of serum neuropeptide levels and their relationship to cognitive impairment and psychopathology in male patients with chronic schizophrenia

**DOI:** 10.1038/s41537-023-00425-1

**Published:** 2024-01-03

**Authors:** Wenxi Sun, Tingting Jin, Haidong Yang, Jin Li, Qing Tian, Ju Gao, Ruijie Peng, Guangya Zhang, Xiaobin Zhang

**Affiliations:** 1 Suzhou Medical College of Soochow University, Suzhou, 215031 Jiangsu China; 2 Psychiatry Department of Suzhou Guangji Hospital, Affiliated Guangji Hospital of Soochow University, Suzhou, 215137 Jiangsu China; 3 Department of Psychiatry, The Fourth People’s Hospital of Lianyungang, The Affiliated KangDa College of Nanjing Medical University, Lianyungang, 222003 PR China

**Keywords:** Transporters in the nervous system, Schizophrenia

## Abstract

Serum neuropeptide levels may be linked to schizophrenia (SCZ) pathogenesis. This study aims to examine the relation between five serum neuropeptide levels and the cognition of patients with treatment-resistant schizophrenia (TRS), chronic stable schizophrenia (CSS), and in healthy controls (HC). Three groups were assessed: 29 TRS and 48 CSS patients who were hospitalized in regional psychiatric hospitals, and 53 HC. After the above participants were enrolled, we examined the Repeatable Battery for the Assessment of Neuropsychological Status (RBANS) and the blood serum levels of α-melanocyte stimulating hormone (α-MSH), β-endorphin (BE), neurotensin (NT), oxytocin (OT) and substance.P (S.P). Psychiatric symptoms in patients with SCZ were assessed with the Positive and Negative Syndrome Scale. SCZ patients performed worse than HC in total score and all subscales of the RBANS. The levels of the above five serum neuropeptides were significantly higher in SCZ than in HC. The levels of OT and S.P were significantly higher in CSS than in TRS patients. The α-MSH levels in TRS patients were significantly and negatively correlated with the language scores of RBANS. However, the BE and NT levels in CSS patients were significantly and positively correlated with the visuospatial/constructional scores of RBANS. Moreover, the interaction effect of NT and BE levels was positively associated with the visuospatial/constructional scores of RBANS. Therefore, abnormally increased serum neuropeptide levels may be associated with the physiology of SCZ, and may cause cognitive impairment and psychiatric symptoms, especially in patients with TRS.

## Introduction

According to the World Health Organization, schizophrenia (SCZ) is among the most impactful of major psychiatric diseases. Particularly, SCZ is estimated to affect about 1% of the worldwide population, with an age-standardized prevalence of 287.4 per 100,000 population, and is ranked as a major cause of disability due to mental disorders^1,2^. With population growth and aging, the burden of SCZ-induced illness continues to increase, especially in middle-income countries^3^. Patients with SCZ tend to have poor eating habits and experience weight gain, with male patients favoring smoking and co-morbid substance use, all of which contribute to a 13–15 year reduction in their life expectancy^4^. The main symptoms of the disease are considered both positive (e.g., delusions and hallucinations) and negative (e.g., blunted affect and social withdrawal) and include the loss of cognitive functions (e.g., memory and executive functions)^5–7^. A recent review of SCZ has concluded that so-called first-degree symptoms are not as important and that cognitive dysfunction is the key clinical symptom of the disorder^8^. Cognitive deficits not only occur during the acute onset of the disease but persist throughout the disease, especially in the chronic phase, leading to severe cognitive impairment^9^. Previous studies^10,11^ have found deficits observed in patients with SCZ in all domains of neuropsychological functioning, with deficits in executive function, memory and sustained attention being particularly prominent.

Despite the severity of cognitive impairment in patients with SCZ, current clinical treatment of SCZ remains primarily focused on the immediate reduction or suppression of psychotic deterioration, with the goal of improving general symptoms as well as improving basic life skills^12^. Antipsychotic drugs are primarily used in the pharmacological treatment of SCZ. A substantial amount of trial data supports the conclusion that these medications can reduce symptoms, particularly positive symptoms, and also to a limited extent negative symptoms, and thus enhance cognitive functioning^13^. However, these drugs also place a significant somatic burden on the patient, including sedation, weight gain, and especially extrapyramidal symptoms^14^. Despite the fact that antipsychotic drugs have been routinely used to treat SCZ for decades, over one-third of SCZ patients do not respond to therapy^15,16^. There is considerable evidence to support the hypothesis that these schizophrenic patients who do not respond well to medications appear to differ from others in terms of pathological processes and outcomes, particularly in terms of cognitive functioning^17,18^. The American Psychiatric Association defines treatment-resistant SCZ (TRS) as the presence of little or no symptom decrease after a 6-week trial of at least two antipsychotics at a therapeutic dose for a sufficient period of time^19^. In other words, the treatment of TRS is difficult. Even with the use of clozapine, which has a multi-receptor mechanism of action, little improvement is seen^20^. Therefore, there is a need to identify new pathological mechanisms to facilitate treatment of TRS with more objective and effective treatment options.

The development of antipsychotic drugs originated from the dopamine (DA) hypothesis more than 50 years ago^21–23^. According to the DA hypothesis, SCZ symptoms are caused by an imbalance in DA^24^. However, as clinical research has progressed, no single dysfunction or impairment has been found in patients’ brains which can fully explain the etiology of SCZ^25^. In addition to monoamines such as DA, neuropeptides are one of the most active and representative classes of neurotransmitters, consisting of neuroactive peptides involved in brain processes such as membrane excitability, synaptogenesis, local blood flow, glial cell structure, and so on^26,27^. Neuropeptides are produced in the cytoplasm of neurons, are stored in vesicles, and are released from vesicles upon electrical activation of neurons. They are broadly dispersed throughout the brain and are frequently co-localized and co-released with monoamine neurotransmitters like DA, glutamate, or γ-aminobutyric acid^28,29^. Neuropeptides exhibit reciprocal neurotransmitter connections or independent activity in preclinical animal models by colocalizing with traditional monoaminergic transmission^30,31^. According to studies in humans and animal models^32,33^, the pathophysiology of psychiatric disorders like SCZ may involve deficits in neuropeptide signaling, resulting in disrupted dopaminergic neurotransmission in mesostriatal and mesocorticolimbic circuits that are highly relevant to SCZ. Accordingly, reversal of abnormalities in neuropeptide signaling pathways may be a novel approach to treating SCZ^34,35^.

Current research on neuropeptides has focused on α-melanocyte stimulating hormone (α-MSH), β-endorphin (BE), neurotensin (NT), oxytocin (OT) and Substance.P (S.P), and their specific distribution and functions are presented in Table 1. α-MSH has been linked to anorexic function as well as systemic (i.e., peripheral and central) anti-inflammatory and cytoprotective mechanisms^36,37^, and it has also been linked to the metabolic syndrome in patients with psychiatric disorders^38^. BE, which is derived from agranulocortinogen along with α-MSH, are endogenous reinforcing mediators that increase midbrain limbic dopaminergic neurotransmission, and these findings suggest a possible opioid-DA interaction^39,40^. Furthermore, given the role of BT in regulating the stress response, many psychiatric disorders can be attributed (at least in part) to some abnormality in BE levels^41,42^. Moreover, BE is a peptide that acts throughout the body, especially in the brain.

**Table 1:** Main sources and major functions of serum neuropeptides.

Serum neuropeptide	Main sources	Major function
α-MSH	Distal part of the pituitary gland and extra-glandular cells	Anti-inflammatory effect Anti-bacterial effect Contributes to innate immunity Suppressed food intake
β-Endorphin (BE)	The arcuate nucleus of the hypothalamus (ArN), the anterior and neurointermediate lobes of the pituitary gland and the nucleus tractus solitaries	Analgesic effect Immunosuppressive effects Behavior Moderation Modifies the sleep–wakefulness cycle
Neurotensin (NT)	The arcuate nucleus and parvocellular paraventricular nucleus	Stimulation of gonadotropin-releasing hormone, dopamine, growth inhibitory hormone and adrenocorticotropin-releasing hormone secretion
Oxytocin (OT)	The supraoptic and paraventricular nuclei of the hypothalamus; extra-hypothalamic locations, including corpus luteum and suggestively, in adrenal tissue and testis	Enhances uterine contractions during labor, promotes lactation, inhibits steroid production, and stress response regulation
Substance.P (S.P)	The substantia nigra, hypothalamus, limbic system, base of the IV ventricle, ganglion cell bodies, pituitary gland, pineal gland, dorsal horn of the spinal cord, posterior roots of the spinal nerves, and cutaneous nerves	Analgesic effect Neurogenic inflammatory reaction Involved in regulating sensation, movement, emotion

Also widely distributed in the human brain and in the periphery is NT, which regulates neural circuits involved in SCZ^43^. Activation of NT receptors (NTS1) in the ventral tegmental area, for example, increased activity of mesocortical DA neurons, the creation of which brain circuits is connected with cognitive deficiencies in SCZ^44^. Evidence suggests that the centrally acting, peripherally given NT analog PD149163 can cure psychosis and anxiety in animal models^45^. Another neuropeptide that is associated with cognitive impairment in psychiatric disorders is OT. Acute OT treatment has been demonstrated in certain studies to enhance cognitive performance in people with SCZ^46,47^. In addition, Fiefel et al. (2012)^48^ discovered substantial gains in OT connected with short-term and long-term verbal memory, but not with working memory. Finally, another neuropeptide which like NT acts in both the central and peripheral nervous system is S.P. On the one hand, S.P can influence various central nervous system processes, including emotional behavior, stress, anxiety and depression;^49^ on the other hand, S.P, like BE, is embedded in the integration of stressful emotional reactions^50^. However, the relationship between S.P and SCZ is currently unclear and needs to be further explored.

Contrary to conventional assumption, males are somewhat more likely than women to suffer from SCZ^8^. Similarly, males outnumber women in China as long-term inpatients with SCZ, and male patients have earlier onset and more severe clinical symptoms (particularly cognitive impairment)^51,52^. Furthermore, sex hormonal influences on cognitive performance could potentially confound our results^53^. However, there are no published studies on neuropeptides in Chinese chronic male schizophrenic patients (especially with TRS). We hypothesized, as previously stated, that neuropeptides (including α-MSH, BE, NT, OT and S.P) may be related to pathological mechanisms in chronic patients with SCZ (especially TRS). Thus, the present study aimed to investigate (1) whether the levels of the above five neuropeptides are variable in chronic patients with SCZ, especially in TRS; (2) whether cognitive functions are variable in chronic patients with SCZ, especially in TRS; (3) whether there is an association between the levels of the above five neuropeptides and cognitive functions in chronic patients with SCZ, especially in TRS; and (4) whether certain neuropeptides are associated through interactions with cognitive function in chronic SCZ patients, especially those with TRS.

## Results

### Sociodemographic features

In this study, the participants were divided into three groups: a “TRS Group”, comprising 29 treatment-resistant male SCZ patients, a “CSS Group”, comprising 48 chronic stable SCZ patients, and an “HC Group”, comprising 53 healthy male participants. There was no statistically significant difference among the three groups in age, years of education, BMI and smoking status (all *p* > 0.05). Additionally, there were no discernible differences between the TRS and CSS groups in terms of age of onset, duration of illness or dose of CPZ equivalent (all *p* > 0.05). However, there were notable discrepancies in the PANSS total and subscale scores between the TRS and CSS groups (all *p* < 0.05; Table 2).

**Table 2:** Demographic and clinical data of TRS patients, CSS patients and HC.

	TRS(*n* = 29)	CSS(*n* = 48)	HC(*n* = 53)	*F*/*χ*^2^	*p*
Age (years)	40.55 ± 9.11	40.81 ± 10.22	40.62 ± 9.92	0.008	0.992^a^
Years of education	8.55 ± 2.96	9.31 ± 3.31	9.47 ± 2.99	0.867	0.423^a^
BMI (kg/m^2^)	24.52 ± 3.74	24.49 ± 3.46	25.19 ± 3.03	0.666	0.515^a^
Smoking	15 (51.72%)	26 (54.17%)	23 (43.40%)	1.262	0.532^b^
Age of onset(years)	25.90 ± 9.87	27.48 ± 8.22		0.576	0.450^a^
Duration of illness(months)	177. 03 ± 79.88	157.69 ± 106.54		0.713	0.401^a^
Dose of CPZ equivalent	619.66 ± 200.09	575.38 ± 196.78		0.904	0.345^a^
PANSS total	74.07 ± 7.166	48.10 ± 8.03		204.749	<0.001^a^
P subscores	14.28 ± 5.26	9.04 ± 2.17		37.231	<0.001^a^
N subscores	24.86 ± 3.87	13.44 ± 4.75		119.631	<0.001^a^
G subscores	34.72 ± 4.31	25.63 ± 4.21		83.089	<0.001^a^

*BMI* body mass index, *CPZ* chlorpromazine, *PANSS* Positive and Negative Symptom Scale.

^a^One-way analysis of variance.

^b^*χ*^2^ test.

### Cognitive performance in TRS patients, CSS patients and HC

The means and standard deviations of the RBANS scores of TRS patients, CSS patients and HC are shown in Table 3. Both TRS and CSS patients performed worse than HC in total score and all subscales (all *p* < 0.01). After controlling for age, education, BMI and smoking status, the differences remained significant for the RBANS total score and subscale scores (all *p* < 0.01). Even after Bonferroni correction, the difference in cognitive function among the three groups remained significant (Table 3).

**Table 3:** Index scores on the RBANS in TRS patients, CSS patients, and HC.

	TRS(*n* = 29)	CSS(*n* = 48)	HC(*n* = 53)	*F*	*p*
Immediate memory	47.17 ± 12.07	52.23 ± 19.30	80.09 ± 17.43	21.136	<0.001^a^
Visuospatial/constructional	65.79 ± 15.63	71.94 ± 14.77	88.92 ± 15.58	10.237	<0.001^a^
Language	70.07 ± 12.50	73.52 ± 14.54	96.62 ± 12.34	22.405	<0.001^a^
Attention	79.21 ± 12.85	83.96 ± 14.21	106.02 ± 11.42	23.538	<0.001^a^
Delayed memory	52.52 ± 14.66	54.56 ± 16.92	82.75 ± 18.63	18.377	<0.001^a^

*RBANS* the Repeatable Battery for the Assessment of Neuropsychological Status.

^a^*F* value was controlled for age, smoking, BMI and education.

### The levels of serum neuropeptides in TRS patients, CSS patients and HC

**Fig. 1:**
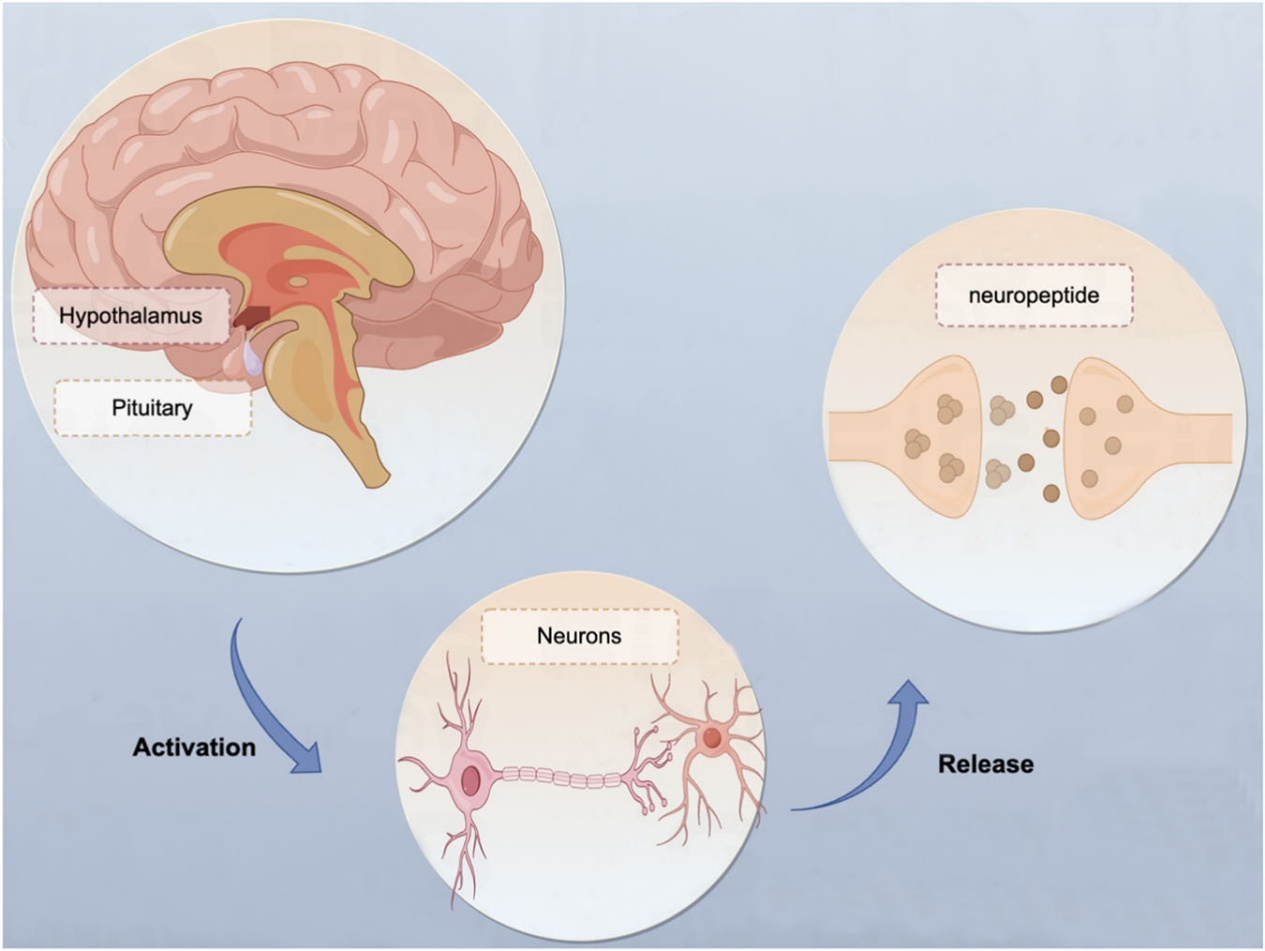
Overview of neuropeptide mechanisms of action. Neuropeptides are synthesized within the cytoplasm of neurons, stored within vesicles, and subsequently discharged upon neuronal electrical stimulation. They exhibit extensive distribution throughout the brain and frequently co-localize and co-release alongside monoamine neurotransmitters, such as dopamine, glutamate, or γ-aminobutyric acid.

The levels of lg serum neuropeptides in TRS patients, CSS patients and HC are shown in Table 4. The lg serum neuropeptide levels, such as lg α-MSH levels and lg BE, lg NT, lg OT and lg S.P levels, were significantly higher in TRS and CSS patients than in HC (all *p* < 0.01). There were considerable differences in the lg serum neuropeptide levels in TRS patients, CSS patients and HC after adjusting for age, years of education, BMI and smoking status (all *p* < 0.01). Except for OT levels, significant differences in the remaining serum neuropeptides among the three groups persisted after application of the Bonferroni correction (Fig. 1).

**Table 4:** Serum neuropeptides in TRS patients, CSS patients and HC.

	TRS(*n* = 29)	CSS(*n* = 48)	HC(*n* = 53)	*F*	*p*
α-MSH	3.61 ± 0.30	3.61 ± 0.33	3.34 ± 0.31	4.441	<0.001^a^
β-Endorphin	3.45 ± 0.28	3.53 ± 0.24	3.22 ± 0.24	8.504	<0.001^a^
Neurotensin	2.89 ± 0.15	2.92 ± 0.15	2.74 ± 0.15	10.584	<0.001^a^
Oxytocin	3.36 ± 0.27	3.41 ± 0.27	3.23 ± 0.29	3.093	<0.007^a^
Substance.P.	2.63 ± 0.35	2.69 ± 0.25	2.35 ± 0.27	8.398	<0.001^a^

^a^*F* value controlled for age, smoking, BMI and education.

The activities of lg OT (*F* = 3.363, *p* = 0.003) showed significant differences, with diagnosis as a fixed variable and age, years of education, BMI, smoking status, age of onset, duration of illness and CPZ equivalency dosage as covariance factors between the TRS and CSS groups.

### Associations between serum neuropeptide levels and cognitive impairment in TRS patients and CSS patients

**Fig. 2:**
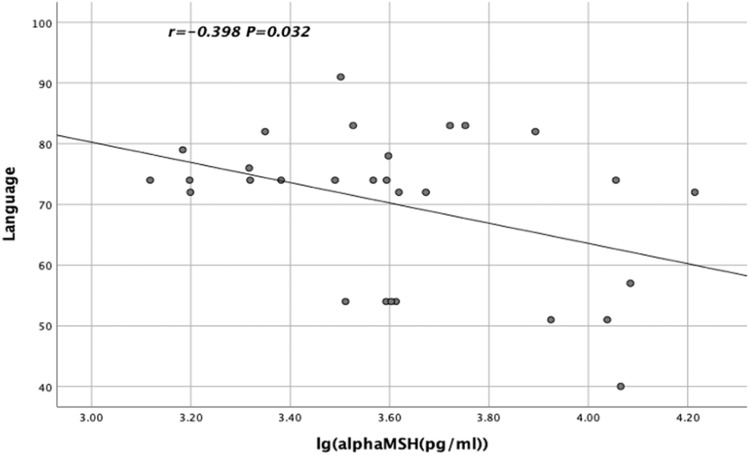
Relationship between lg α-MSH levels and TRS patients' language scores on the RBANS. The lg α-MSH levels in TRS patients showed a significant and negative correlation with the language scores of RBANS (*r* = −0.398, *p* = 0.032).

**Fig. 3:**
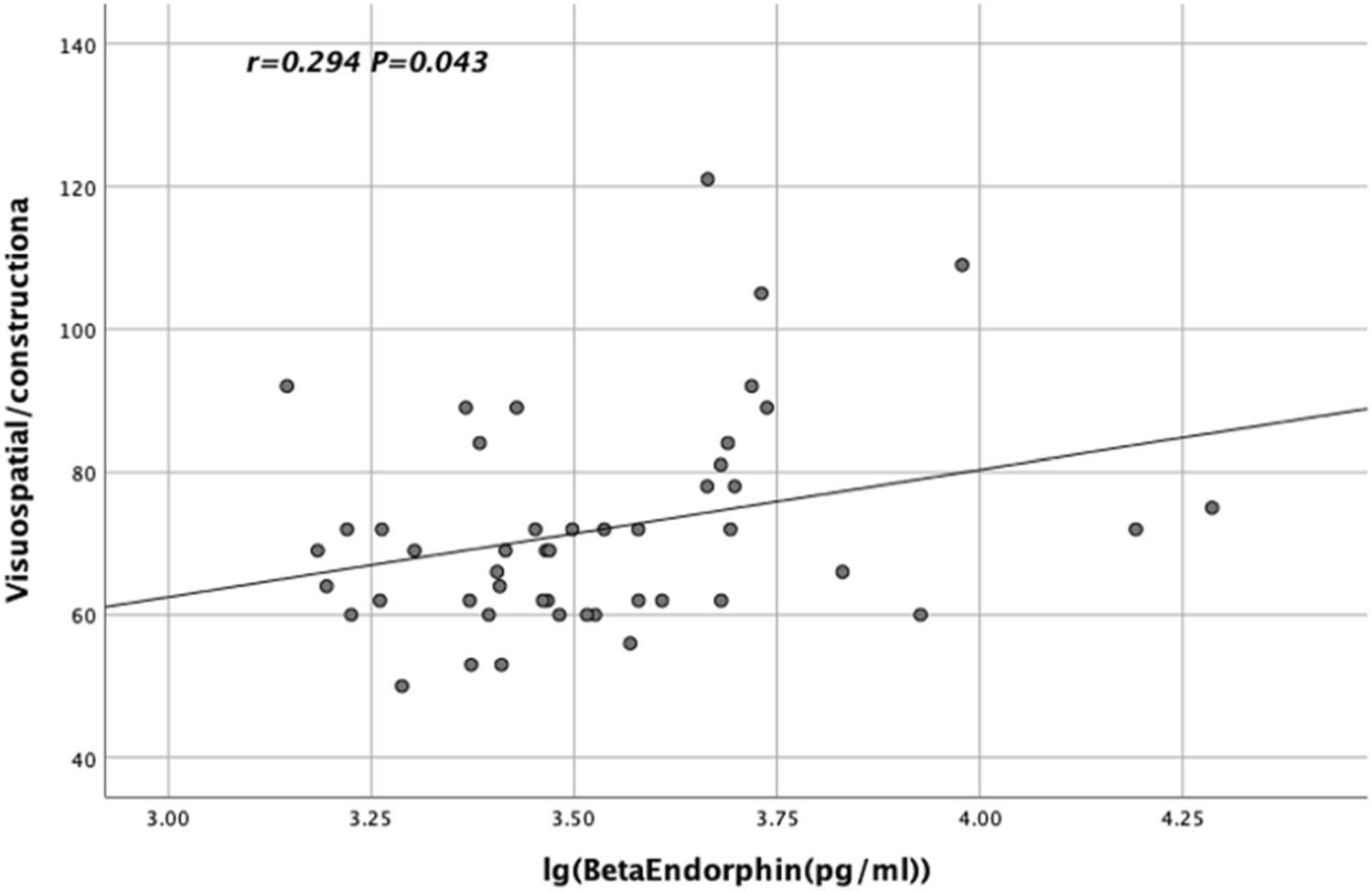
Relationship between lg BE levels and visuospatial/constructional scores on the RBANS in CSS patients. The lg BE levels in CSS patients exhibited a significant and positive correlation with the visuospatial/constructional scores of RBANS (*r* = 0.294, *p* = 0.043).

**Fig. 4:**
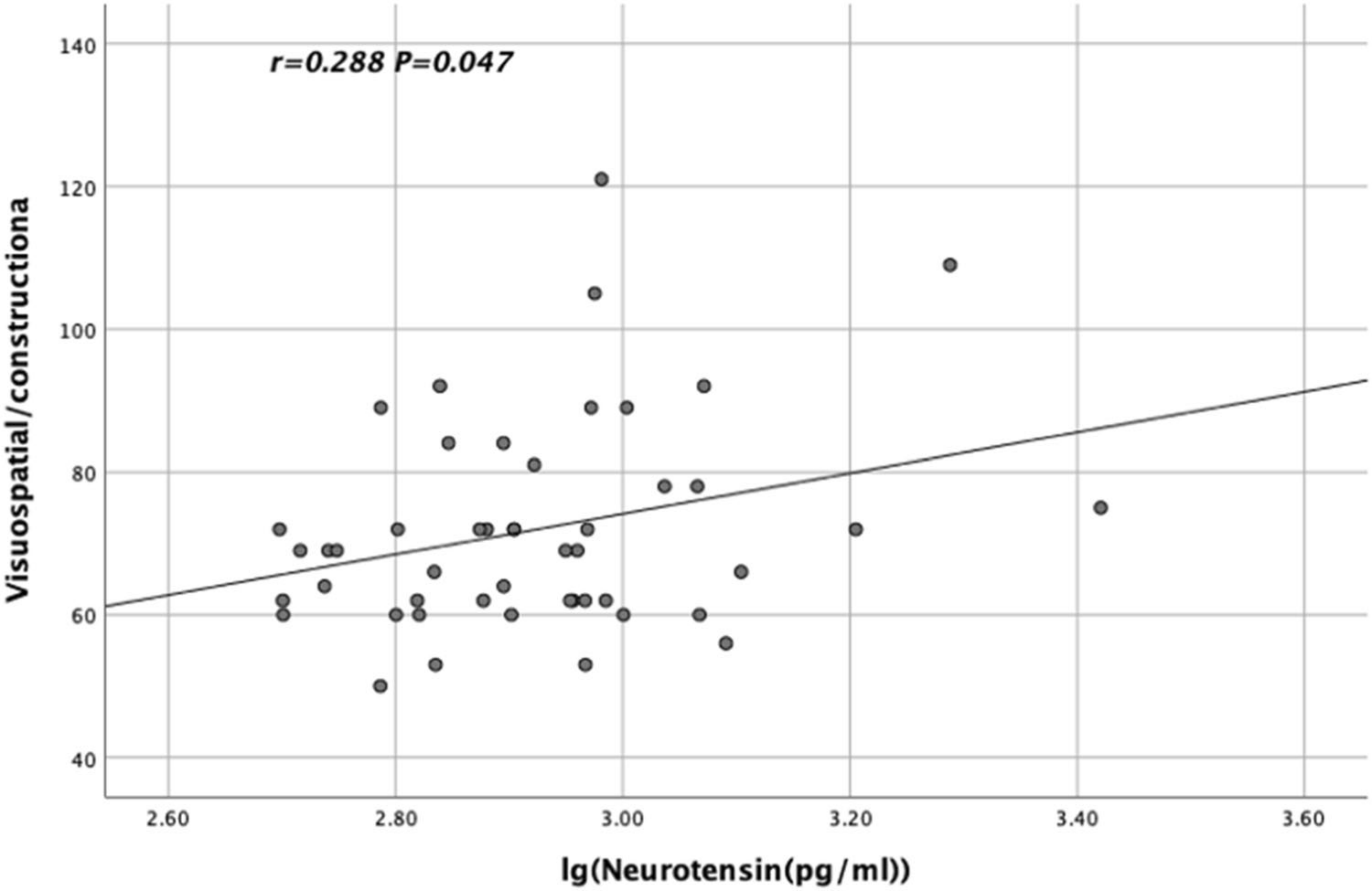
Relationship between lg NT levels and visuospatial/constructional scores on the RBANS in CSS patients. The lg NT levels in CSS patients demonstrated a significant and positive correlation with the visuospatial/constructional scores of RBANS (*r* = 0.288, *p* = 0.047).

The lg α-MSH levels in TRS patients were significantly and negatively correlated with the language scores of RBANS (*r* = −0.398, *p* = 0.032, Fig. 2). In contrast, the lg BE levels in CSS patients were significantly and positively correlated with the visuospatial/constructional scores of RBANS (*r* = 0.294, *p* = 0.043, Fig. 3). Moreover, the lg NT levels in CSS patients were significantly and positively correlated with the visuospatial/constructional scores of RBANS (*r* = 0.288, *p* = 0.047, Fig. 4).

After controlling for possible influencing factors such as age, years of education, BMI, smoking status, age of onset, duration of illness and CPZ equivalence dose, language scores and lg α-MSH levels were found to be significantly correlated (*R*^2^ = 0.494, *p* = 0.019) in TRS patients by stepwise multiple regression analysis. In addition, after controlling for possible influencing factors such as age, years of education, BMI, smoking status, age of onset, duration of illness and CPZ equivalence dose, visuospatial/constructional scores and lg BE levels were found to be significantly correlated (*R*^2^ = 0.113, *p* = 0.030) in CSS patients by stepwise multiple regression analysis.

### Interaction of lg NT and BE levels

Lg NT levels were significantly and positively correlated with lg BE in the CSS patients (*r* = 0.870, *p* < 0.001). After controlling for age, years of education, BMI, smoking, age of onset, duration of disease and CPZ equivalent dose, partial correlation analysis revealed that lg NT levels were significantly correlated with lg BE levels in the CSS group (*r* = 0.864, *p* < 0.001). Lg NT*BE interaction was found to have a positive correlation with the visuospatial/constructional scores of RBANS when the interaction term between lg NT and BE levels was developed (*r* = 0.299, *p* = 0.039).

## Discussion

The following are the key findings of the current investigation on long-term medicated SCZ inpatients: (1) Serum neuropeptide levels, including α-MSH, BE, NT, OT and S.P, were significantly higher in patients with chronic SCZ on long-term medication than in HC, and patients in the CSS group had substantially higher levels of OT and S.P than the TRS group; (2) patients with chronic SCZ had considerably lower RBANS test scores than HC, and TRS had lower RBANS test scores than CSS, but the differences were not statistically significant. (3) α-MSH levels of TRS patients were significantly and inversely linked with their RBANS language scores; (4) NT and BE levels in CSS patients were significantly and positively correlated with their RBANS visuospatial/constructional scores; and (5) in the CSS group, there was a strong correlation between NT and BE levels. The RBANS visuospatial/constructional scores were found to positively correlate with the NT-BE interaction.

As predicted, levels of the five neuropeptides (α-MSH, BE, OT, NT and S.P) were significantly higher in chronic male SCZ patients than in healthy controls. However, these five neuropeptides have only recently been studied and previous results did not reveal significant differences between schizophrenic patients and HC^54^. Interestingly, there have also been studies showing significantly higher levels of BE in schizophrenic patients^55,56^, and others have concluded that low levels of OT are associated with SCZ^57,58^. Surprisingly, other studies have found that OT levels are high in patients with SCZ^59–61^. Moreover, low NT concentrations in the cerebrospinal fluid were linked to more severe psychopathology in SCZ, such as an increase in cognitive difficulties, delusions and hallucinations^62,63^, but changes in NT concentrations in the peripheral blood are likely to vary. Furthermore, a preclinical investigation utilizing a multiplex immunoassay found elevated NT and OT in hereditary and/or environmental SCZ-like rats^64^. In contrast, most of the previously published studies that contradict our findings had limitations such as small sample sizes, heterogeneity or short observation periods, which weakened their reliability. Taken together, findings regarding neuropeptide levels in patients with SCZ have been inconclusive and more research is needed to identify their role in SCZ.

In addition, our results showed that most of the neuropeptide levels in CSS were higher than in TRS, especially for OT and S.P. To the best of our knowledge, no studies have thus far explored the differences in neuropeptide levels between TRS and CSS. However, professional research on TRS has been undertaken^65–68^. Based on the results of previous studies with SCZ, schizophrenics can be grouped into a variety of patient segments^69^. Specifically, those with SCZ have diverse phenotypes, which are characterized by different indicators and symptoms of illness as well as life course, with various risk factors leading to disease, including a complex genetic load capacity, a broad spectrum of neurobiological characteristics implicating a pathophysiology of structure and function that is not shared by all patients, and a mixed range of expressed responses to treatment^70^. This demonstrates the heterogeneity which is characteristic of SCZ. It does, however, suggests new pathways involved in the pathogenesis of TRS. Compared with other patients with severe mental illness, TRS patients are difficult to treat, have poor social function recovery^71^, experience a heavy social and family economic burden^72^ and suffer from poor prognosis^16^. Furthermore, first-degree relatives of TRS have been shown to be at higher risk for SCZ compared with non-TRS patients and healthy controls^73^. In addition to hereditary variables and clinical symptoms, recent systematic studies have demonstrated that TRS patients have reduced gray matter, especially in the frontal lobe, increased white matter volume, and decreased striatal DA production compared to non-TRS patients^74^. Striatal synaptic DA has been implicated in the antipsychotic response, with 50% occupancy of D2 DA receptors required to alleviate clinical symptoms^75^. Similarly, we predicted that neuropeptides which co-localize and co-release with DA would endure similar modifications. Nevertheless, there is currently a lack of sufficient data to support this hypothesis. Further research may possibly establish that TRS has distinct neuropsychological traits.

In the present research, there is no doubt that cognitive function was significantly impaired in patients with SCZ who take antipsychotic drugs for a long period. This conclusion is consistent with a wealth of previous research evidence^76–79^. In terms of disease types, compared with other severe mental disorders, cognitive problems in patients with SCZ are most pronounced^80^. Virtually all people with SCZ experience cognitive impairment^81^. From the perspective of clinical symptoms, in comparison to positive symptoms such as hallucinations and delusions, cognitive function has been recognized as a therapy priority. While such recognition has been relatively late in coming, it is very crucial for SCZ patients’ long-term recovery^81^. For recovery from SCZ, cognitive deficiencies are apparent at the outset of disease^82^, must be endured into old age^83^, and are resistant to the effects of traditional medication for symptoms. Specifically, SCZ has been linked to substantial abnormalities in reasoning, planning, abstract thought and problem-solving, as well as impairments in working memory, attention, processing speed, and visual and linguistic acquisition^84–86^. However, studies in the field of cognitive function in TRS are scarce and findings suggest that cognitive deficits in TRS are more prevalent and severe^87^. Similarly, patients with TRS have been reported to have more prominent cognitive deficits in language domains, such as in speech, verbal intelligence, verbal fluency and verbal memory^88^. In contrast to the above findings, other authors have reported low scores on a nonverbal memory test of the Brief Cognitive Assessment of Schizophrenia in TRS patients in a cross-sectional study^89^. In a longitudinal study with at least 5 years of clinical follow-up, their findings suggested moderate improvement in cognitive function in TRS over time (Cognitive Index, mean effect size = 0.32)^90^. Nevertheless, overall cognitive deterioration (Cognitive Index, mean effect size = −0.64) was found to be more pronounced in TRS in a between-group comparison. Remarkably, our study was not statistically significant, although we found that the cognitive scores in TRS were lower than in CSS. Perhaps subsequent studies with larger samples will unveil the cognitive functioning domain of TRS.

Consistent with the latest emerging study^54^, we found that the level of cognitive function in schizophrenic patients was not implicated in neuropeptide levels such as S.P and OT. In contrast, our research found that the levels of NT, BE and α-MSH were significantly correlated with cognitive function. Evidence supporting the conclusions of our study has also been provided by Zak et al.^91^, who found that OT was not associated with executive function. Paradoxically, there is also a variety of clinical evidence pointing in the opposite direction, such as a randomized double-blind placebo-controlled trial^47^ in which OT recipients showed improvements in several cognitive indicators. Moreover, a recent study^92^ found a negative association (*β* = −0.46, *p* = 0.03) between plasma OT levels and cognitive function in patients with SCZ. Similarly, Urban-Kowalczyk et al.^93^ did not find any evidence linking S.P to cognitive function in patients with SCZ. Apparently, there has been little evidence reported regarding S.P and cognitive function in schizophrenic patients, and more evidence is required to confirm our conclusions.

Unlike S.P, which has received little attention, researchers have performed a great deal of work on BE and its relevance to the psychiatric field. The hypothesis of endogenous “hypermorphinergic pathology” has been proposed, suggesting that BE concentrations are significantly elevated in schizophrenic patients^94^. Increased BE levels are hypothesized to affect DA function, a precursor to endogenous opioids. Presynaptic opioid μ receptors, which also reduce DA pathway inhibition, enhance DA release^95^. Patricia Goldman-Rakic’s groundbreaking research^96^ revealed the neural circuitry required to generate mental representations in the dorsolateral prefrontal cortex for working memory, and she found that DA had a dramatic effect on this area of the brain, with DA depletion leading to severe impairment of working memory. Hence, enhancing beneficial behaviors with DA may be a reasonable way to improve cognitive deficits. Thus, elevated BE levels may improve cognitive deficits by affecting the DA pathway. Another neuropeptide that interacts closely with dopaminergic interactions is NT, and its mechanism of action has been extensively studied. In the nucleus ambiguus, NT receptors are co-localized with presynaptic and postsynaptic DA receptors. The binding effect of NT at these locations can be summarized as an antagonistic effect on D2 receptors. Notably, Fawaz et al.^97^ found that treatment with NT significantly enhanced DA release from the vomeronasal nucleus in rat brain slices during pulse string stimulation. Accordingly, we hypothesize that similar to BE, NT may have a similar effect on cognitive function. As expected, we discovered that the interaction of BE and NT was positively correlated with cognitive function. Consequently, we hypothesize that NT may affect cognitive function directly through the DA system or that NT may indirectly affect cognitive function through its interaction with BE.

In contrast to CSS, we found a significant correlation between α-MSH and cognitive function in TRS. However, similar studies are scarce, and a recent study^54^ has come to the opposite conclusion from our study. We currently have an understanding of the mechanisms underlying the role of α-MSH and the DA system in feeding behavior^98^, and other investigators have explored the role of α-MSH autoantibodies (autoAbs) in impaired cognitive function^99^. It has also been suggested that altered production of autoAbs which react with glycopeptides and α-MSH may be associated with Alzheimer’s disease-related peptidergic dysregulation. Increased levels of serum complex formation are associated with increased inhibition of α-MSH-mediated behavior compared to free α-MSH autoAbs^100^. As a result, elevated total and complexed galanin autoAbs may reflect enhanced inhibition of central galanin signaling in Alzheimer’s. Total and complexed IgG autoAb levels of galanin correlate positively with the Mini-mental State Examination score, showing that higher inhibition of galanin signaling may be associated with improved cognitive function^101^. Unfortunately, to date, the above hypothesis has not been confirmed in SCZ, and we could not verify that the correlation between α-MSH and cognitive function is yet another feature of TRS. However, it is likely that α-MSH will become a new approach to elucidate the pathological mechanism of schizophrenia.

There are several limitations that should be considered in the present research. First, our participants were on long-term antipsychotic medication, and we did not exclude the effect of long-term medication on neuropeptide levels. Second, our study was conducted with male subjects, and the findings need to be validated in the female population. Third, the sample sizes of our TRS and CSS patients were small, and the validity of our findings needs to be verified in a larger sample. Fourth, as a cross-sectional study, our findings have not been dynamically observed over time. Finally, although we observed some correlations between neuropeptide levels, cognitive function and treatment resistance, the underlying mechanisms remain to be investigated.

## Conclusion

In conclusion, our research preliminarily demonstrates that chronic patients with SCZ have significantly higher serum neuropeptide levels compared to HC, including α-MSH, BE, NT, OT and S.P, while TRS patients have lower neuropeptide levels than CSS patients. Unexpectedly, cognitive function was significantly worse in chronic schizophrenic patients comparted to HC. In addition, the level of α-MSH in TRS was significantly and negatively correlated with cognitive function. BE and NT levels in CSS were significantly and positively correlated with cognitive function, and an interaction between BE and NT levels was observed. Considered together, we hypothesize that serum neuropeptides may have a specific modulatory action on particular aspects of cognitive function in SCZ. The mechanisms underlying the relationship between serum neuropeptides and SCZ deserve detailed investigation.

## Material and methods

### Participants

This study employed an observational, cross-sectional, retrospective design with a case-control approach, conducted from October 2018 to October 2020. A total of 29 patients diagnosed with TRS and 48 patients with chronic stable schizophrenia (CSS) were selected from of SCZ inpatients (all male) in local mental hospitals, specifically Lianyungang Fourth People’s Hospital. All patients met the following inclusion criteria: (1) aged 18–60 years old, Han Chinese; (2) diagnosed with schizophrenia based on the Structured Clinical Interview for the Diagnostic and Statistical Manual of Mental Disorders, 4th edition (DSM-IV); (3) had a minimum illness duration of 2 years; and (4) received consistent administration of neuroleptic medications at stable doses for a minimum of 12 months, and (5) completed at least elementary school and demonstrated the ability to comprehend the questions posed by the investigators. Using accepted methods, antipsychotic drugs, including first- and second-generation antipsychotics, were translated into roughly similar daily milligram doses of chlorpromazine (CPZ) for each individual^102^. Exclusion criteria: first, patients who presented with somatic disorders or substance dependence were excluded. Second, patients who had experienced a major life event (e.g., divorce, widowhood, etc.) in the month prior to admission were excluded. In addition, patients who had not completed primary education were excluded from the study.

The local population of Lianyungang City was selected for the recruitment of 53 HC, all of whom were men and matched for age, education and BMI. To evaluate current mental state and any personal or family history of mental disorders, unstructured interviews were conducted. None of the healthy subjects presented a personal or family history of psychiatric disorders. Both the patients and control participants provided comprehensive medical histories (including use of sleep aids and antipsychotics), along with the results of physical examinations (e.g., vital signs, cardiopulmonary function, etc.) and laboratory tests (e.g., blood cell analysis, electrolytes, liver and kidney function, etc.). We thus ensured the inclusion of participants with normal results for these tests in our study. Subjects suffering from serious medical conditions were barred from participating. All healthy subjects were from the same ethnicity and geographic region as the patient group. A psychiatrist completely described the research protocol and procedures to each patient before they gave their written informed consent to participate in the study. The information regarding the study was conveyed in a manner that maximized comprehension, taking into account the subjects’ level of understanding and emotional readiness. In some cases, the researcher provided a detailed description to both the subjects and their parents or guardians. The Lianyungang Fourth People’s Hospital’s Institutional Review Board gave its approval to the research protocol. All methods were performed in accordance with the Declaration of Helsinki.

### Defining TRS and CSS

The following criteria have been used in several earlier research studies to define TRS^103–106^: ineffectiveness of continuous treatment with two antipsychotic medications at an adequate dose for a duration exceeding 6 months; a minimum daily dose of chlorpromazine (CPZ) or its equivalent, equal to or greater than 600 mg; and a total score on the Positive and Negative Syndrome Scale (PANSS) of 70 or higher, with each of the eight individual PANSS items (P1, P2, P3, N1, N4, N6, G5, and G9) scoring at least 3. Likewise, the following criteria were used to define CSS based on the findings of numerous prior investigations^103–106^: continuous treatment with a single antipsychotic medication for a minimum of 6 months; CPZ or equivalent daily dose less than 600 mg; and a total PANSS score below 60, with each of the PANSS items described above scoring less than 3.

### Application and scales

The diagnosis of those in the patient groups was first made by a DSM-IV-oriented clinical interview conducted by an experienced psychiatrist, and a sociodemographic data form was completed. Simultaneous assessment of disease symptoms and cognitive functions was performed on the first day of hospitalization (or within the first 3 days of hospitalization if the patient needed to adapt to the tests). In order to avoid the possible influence of medications (e.g., sleep aids) on the patients’ condition, we scheduled the assessment of psychiatric symptoms and cognition for the afternoon.

A sociodemographic and clinical data form was administered to collect sociodemographic data and disease history data from patients and control group participants. In the case of control group participants, the completion of the data form occurred subsequent to confirmation of the absence of any psychiatric diagnosis through a DSM-IV-oriented clinical interview. To evaluate cognitive functions, the Repeatable Battery for the Assessment of Neuropsychological Status (RBANS) was utilized. The PANSS assesses positive and negative symptoms and general psychopathology in patients with psychiatric disorders and measures the severity of these symptoms. Two psychiatrists who had concurrently attended a training session in the use of the scale before the trial started independently assessed the psychopathology of the patients using the PANSS^107^ on the day of blood collection. Two separate PANSS scale interviews were conducted for each patient by the two psychiatrists after the training, guaranteeing the reliability and consistency of the obtained ratings throughout the study. In addition, replication of the PANSS total score assessment maintained an inter-rater reliability correlation coefficient >0.8. The two PANSS total scores of the same patient who met the requirements after enrollment were averaged as their final clinical symptom score.

### Cognitive assessments

In this study, the Chinese version of RBANS^108^ was used to evaluate the cognitive abilities of SCZ patients and healthy controls. Given that RBANS takes about 30 min to complete and has demonstrated strong reliability and validity in individuals with psychosis, it was practical and realistic to employ it in a clinical setting. The cognitive domains under scrutiny encompassed five age-adjusted index scores, namely, the Immediate Memory Index, Visuospatial/Constructional Index, Language Index, Attention Index, and Delayed Memory Index, along with a composite total score. The initial neuropsychological variable scores were transformed to T-scores employing the established criteria provided in the relevant manuals. It is important to note that a higher RBANS score corresponds to an enhancement in cognitive performance.

### Collection and analysis of biological samples

To guarantee a fasting state, all study participants fasted for 8 h prior to blood sampling, and between 7:00 and 9:30 a.m., 5 ml of elbow venous blood were drawn from each participant. The serum samples were kept frozen in a −80 °C refrigerator after the blood samples were centrifuged at 3000 r/min for 15 min to separate the upper serum layer.

Serum neuropeptides were measured using the Bio-Plex MAGPIX System (Human Neuropeptide Magnetic Bead Panel, item number HNPMAG-35K, Bio-Rad), a suspension microbead microarray platform based on the Luminex liquid phase suspension chip. Serum neuropeptides were expressed as one trillionth of a gram per milliliter plasma (pg/ml).

### Statistical analysis

The study data were evaluated using the Statistical Product and Service Solutions 26.00 programme. After descriptive and frequency analysis, the groups were compared. Then, we examined the normality of the data using Q–Q plots and the Kolmogorov–Smirnov test. Given the non-normal distribution of serum neuropeptide levels among patients diagnosed with TRS, CSS, and HC (confirmed by the Kolmogorov–Smirnov test, all *p* < 0.05), we employed a logarithmic transformation (base 10) to convert the serum neuropeptide levels into normally distributed data. Categorical variables were evaluated by the chi-square test, while continuous variables were compared using Student’s *t* test or one-way analysis of variance. Data are presented as means ± standard deviation. All *p* values are two-tailed and the significance level was set at 0.05.

First, using diagnosis as a fixed factor and age, years of education, BMI and smoking status as covariates, multivariate analysis of covariance (MANCOVA) was carried out to compare differences in serum neuropeptide levels and RBANS scores (dependent variables) among TRS patients, CSS patients and HC. Second, using diagnosis set as a fixed factor and age, years of education, BMI, smoking status, age at onset, duration of illness and CPZ equivalence dose as covariates, analysis of covariance (ANCOVA) was used to compare differences in serum neuropeptides levels (dependent variables) between the TRS and CSS groups. In addition, multiple testing was adjusted with the Bonferroni correction (0.05/5). Third, Pearson’s correlation coefficient was used for correlation analysis. Finally, after adjusting for confounding variables such age, years of education, smoking status, BMI, age of onset, length of illness and CPZ equivalent dose, exploratory multiple regression analysis was used to determine the connection between serum neuropeptide levels and RBANS scores.

### Supplementary information

reporting-summary

editorial-policy-checklist

## Data Availability

The data supporting the results of this study are available upon request from the corresponding author.
